# Adverse drug events in Chinese pediatric inpatients and associated risk factors: a retrospective review using the Global Trigger Tool

**DOI:** 10.1038/s41598-018-20868-2

**Published:** 2018-02-07

**Authors:** Huan-huan Ji, Lin Song, Jian-wen Xiao, Yu-xia Guo, Ping Wei, Ting-ting Tang, Xiao-jiang Tian, Xue-wen Tang, Yun-tao Jia

**Affiliations:** 1 Department of Pharmacy, Ministry of Education Key Laboratory of Child Development and Disorders, China International Science and Technology Cooperation Base of Child development and Critical Disorders, Chongqing Key Laboratory of Pediatric, Children’s Hospital of Chongqing Medical University, CHONGQING, 400014 China; 2 Department of Hematology, Children’s Hospital of Chongqing Medical University, CHONGQING, 400014 China; 3 Department of Ear-nose-throat, Children’s Hospital of Chongqing Medical University, CHONGQING, 400014 China; 4 Department of Medical Record, Children’s Hospital of Chongqing Medical University, CHONGQING, 400014 China

## Abstract

Understanding the epidemiology and risk factors of adverse drug events (ADEs) in pediatric inpatient is essential if we are to prevent, reduce or ameliorate the harm experienced. The Global Trigger Tool (GTT) is a method of retrospective medical record review that measures harm in hospitalized children. We employed a three-stage retrospective chart review of random samples of 1800 pediatric inpatients discharged from January 2013 to December 2015. 31 kinds of pediatric-specific triggers were made based on the previous trigger tool studies developed for use in adult or pediatric. Positive predictive value (PPV) of individual triggers, as well as ADEs detection rates were calculated. Stepwise logistic regression was performed to investigate risk factors associated with ADEs. Of 1746 patients, detected in 221 patients (12.7%) with 247 ADEs. The PPV of the trigger tool was 13.3%. Of the 247 ADEs, 82.6% were identified as category E, 11.7% category F and 5.7% category H. The pediatric-focused trigger tool is a feasible and useful tool for detecting pediatric ADEs. Especially for patients who have had more drugs, more doses or more admissions which needs to be closely monitored as triggers to improve the safety.

## Introduction

Adverse drug events (ADEs) are defined by the Institute of Medicine (IOM) as injuries resulting from a medical intervention related to a drug and can manifest as signs, symptoms or laboratory abnormalities^1^. This is more general definition than that provided by the World Health Organization, which defined an adverse drug reaction (ADR) as a response to a drug, which is noxious and unintended, and occurs at doses normally used in man for prophylaxis, diagnosis or therapy of disease, or for the modification of physiologic function^2^. Because this definition of ADR excludes overdose, drug abuse and treatment failure and drug administration errors. An adverse event was defined as any injury (not just that associated with medication or drug use) caused by medical management rather than by the underlying disease or condition of the patient^3^. We only concerned with medication associated adverse events, such as ADR and drug administration errors. ADEs are important causes of iatrogenic morbidity and mortality, especially for children, but the frequency of ADEs is unknown. The reported rates of ADEs range from 0.6% to 20%^4,5^, due to the differences in definitions of ADEs, methods, prescribing habits, age group, and clinical settings. Traditional methods to detect ADEs have focused on voluntary reporting. However, public health researchers have established that on average only 10–20% of errors are ever reported and of those, 90–95% cause no harm to patients^6^. The Global Trigger Tool (GTT) developed by the Institute for Healthcare Improvement (IHI) is a retrospective review of a random sample of inpatient hospital records using “trigger” to identify possible adverse events^6^. The GTT, which requires minimal training, appears to increase the rate of ADE detection 50-fold from traditional reporting methods^7^. Using the GTT, Classen *et al*. found at least ten times more confirmed, serious events than tradition methods^8^. A trigger is a clue condition believed to be associated with the occurrence of an adverse event. The triggers themselves represent specific events including the ordering of certain medications (e.g., antidotes, such as Naloxone), the result of certain abnormal laboratory values (e.g. supratherapeutic serum medication concentrations, such as vancomycin), change in clinical status or symptom (e.g., drug-related rash), and abrupt stop orders *et al*.^3^. In recent years, the GTT has been used in pediatric populations worldwide, such as in the US^9,10^, UK^11^, Norway^12^, Australia^13^, and Japan^4^, has been proved to be reasonable and reliable.

The occurrence of ADEs is associated with both patient characteristics and their health care utilization. With regard to risk factors associated with ADEs in pediatric patients, significant differences have been found in terms of the number of drugs^14^, length of hospital stay^15^, use of antibacterial^15^ and general anesthesia^16^. However, such findings are not always consistent; while some studies found that gender was not significantly associated with ADEs^17^, a study also found that males were at higher ADEs risk compared to females^18^. In addition, little is known about the performance of the GTT and risk factors associated with the occurrence of ADEs in Chinese pediatric inpatients. Practical and reliable methods are needed to identify and detect ADEs in hospital practice. Therefore, The aim of this study was to estimate the frequency of ADEs in Chinese pediatric inpatients in tertiary care hospitals and characteristics associated with the occurrence of ADEs.

## Results

### Patients Characteristics

A total of 1800 patients involved in 1800 cases were identified, of which 54 were excluded including 28 without drug exposure and 26 diagnosed with cancer. Among the final records of 1746 cases, 1135 (65%) were from males and 611 (35%) from females. The age range was between 0.08~17.75 years old with a mean of 3.84, the average length of hospital stay was 7.83 ± 5.28 days (1~63 days), average drugs per patient were 14 ± 7 (1~64), and doses per patient was 114 ± 105 doses (1~1206 doses). Among those 221 patients who had ADEs, 199 (11.4%), 18 (1.0%), 4 (0.2%) patients had one, two and three or more ADEs, respectively. There were no significant differences in age, gender, number of medical diagnoses, admission in the last year, type of admission, department or surgical operation (p > 0.05), whereas significant differences were identified in the length of stay, antibacterial use, number of drugs, doses, triggers and number of admissions between patients with and with no ADEs (*P* ≤ 0.01) (Table 1).

**Table 1:** Characteristics of Patients with ADEs and with no ADEs.

Characteristics	Total (n = 1746)	Patients with no ADEs (n = 1525)	Patients with ADEs (n = 221)	*p*
Age (y)^a^	3.84 ± 3.89	3.86 ± 3.85	3.72 ± 4.12	0.614
Female ratio (%)^a^	35.0% (611/1746)	35.3% (539/1525)	32.6% (72/221)	0.451
Length of stay (d)^b^	7.83 ± 5.28	7.48 ± 4.66	10.23 ± 8.03	<0.001
Drugs per patient^b^	14.18 ± 6.77	13.51 ± 6.10	18.82 ± 9.02	<0.001
Doses per patient^b^	113.94 ± 104.97	102.92 ± 81.30	189.98 ± 187.00	<0.001
Number of triggers^b^	1.34 ± 1.36	1.14 ± 1.19	2.73 ± 1.73	<0.001
Number of medical diagnoses^a^	2.97 ± 1.89	2.83 ± 1.71	2.92 ± 1.98	0.512
Number of admission^b^	1.81 ± 1.42	1.77 ± 1.39	2.07 ± 1.60	0.010
Number of admission in the previous 1 year^a^	0.49 ± 1.04	0.47 ± 1.04	0.61 ± 1.01	0.069
Type of admission^b^				
Elective	1555 (89.1%)	1365 (89.5%)	190 (86.0%)	0.133
Emergent	191 (10.9%)	160 (10.5%)	31 (14.0%)	
Surgical operation^b^				
Yes	506 (29.0%)	442 (28.8%)	64 (30.3%)	0.635
No	1240 (71.0%)	1083 (71.2%)	157 (69.7%)	
Department^b^				
Internal	1080 (61.9%)	931 (61.0%)	149 (67.4%)	0.075
Surgery	666 (38.1%)	594 (39.0%)	72 (32.6%)	
Antibacterial use^a^				
Yes	906 (51.9%)	764 (50.1%)	142 (64.3%)	<0.001
No	840 (48.1%)	761 (49.9%)	79 (35.7%)	

Chi-square test or t-test: ^a^*p* > 0.05, ^b^*p* < 0.01.

### Triggers

We established 31 kinds of triggers, among which 24 were positive (77.4%) during the chart review, and 23 associated with ADEs. Among the final 1746 cases, 1213 (69.5%) had positive triggers. A total of 2291 triggers were detected resulting in a mean rate of 1.3 triggers per patient. Trigger Positive predictive value (PPV) is the number of times a specific trigger independently identified an ADE divided by the number of times a trigger was identified positive. The overall PPV of the aggregate Children’s Hospital of Chongqing Medical University (CHCMU) trigger list was 13.3%. The number of positive triggers, ADEs and PPV for each trigger are displayed in Table 2.

**Table 2:** The Trigger Items of CHCMU and its PPV.

No	Triggers	Positive Triggers	ADEs ^†^	PPV ^‡^ (%)
Laboratory Index				
L1	Partial thromboplastin time (PTT) > 100 s^1,9,23^	23	4	17.4
L2	International normalized ratio (INR) > 3.5^1,20^	2	1	50.0
L3	Hypoglycaemia (≤2.8 mmol.L^−1^)^20^	13	5	38.5
L4	Hyperglycaemia (≥7.8 mmol.L^−1^)^12,20^	40	5	12.5
L5	Rising urea or creatinine (>2 × baseline)^12^	3	0	0
L6	Rising alanine aminotransferase or aspartate transaminase or total bilirubin and alkaline phosphatase (>2 × baseline)^1^	115	14	12.2
L7	Hypokalemia (<3 mmol.L^−1^)^1,12^	32	6	18.8
L8	Hyperkalemia (>5.5 mmol.L^−1^)^1,20^	89	1	1.1
L9	Hyponatremia (<130 mmol.L^−1^)^1,12,20^	12	1	8.3
L10	Leukocyte count < 3*10^9^.L^−1^^23,24^	13	3	23.1
L11	Platelets count < 50*10^9^.L^−1^^12^	0	0	0
L12	Elevated drug levels: Vancomycin > 30 mg.L^−1^\ Amikacin > 5 mg.L^−1^^1,20^	0	0	0
L13	Phenytoin > 40 mg.L^−1^\ Valproic acid > 150 mg.L^−1^\Phenobarbital > 50 mg.L^−1^\Carbamazepine > 15 mg.L^−1^\Clonazepam > 80 µg.L^−1^\Nitrazepam > 200 µg.L^−1^\Oxcarbazepine > 35 mg.L^−1^\Phenobarbital > 20 mg.L^−1[20]^	0	0	0
L14	Cyclosporin A > 400 µg.L^−1^ (treatment)\> 500 µg.L^−1^ (transplantation of organ)	0	0	0
L15	Elevated drug levels: Digoxin > 2 µg.L^−1^ ^1,23^	0	0	0
L16	Elevated drug levels: Lidocaine > 5 mg.L^−1^ ^23^	0	0	0
L17	Elevated drug levels: Theophylline > 20 g.L^−1^ ^1,23^	0	0	0
Antidotes				
A1	Anti-allergic or Adrenaline use^6^	312	21	6.8
A2	Flumazenil use^6^	45	40	88.9
A3	Naloxone\Nalmefene use^6^	54	23	42.6
A4	Anti-emetic use^6^	303	21	6.9
A5	Ongoing or intermittent laxative use^20^	321	20	6.2
A6	Protamine use^20^	16	2	12.5
A7	Antidiarrheal or Antidiarrheal administration^23^	364	35	9.6
A8	Glutathione use	161	16	9.9
A9	Nystatin and Sodium bicarbonate use	25	25	100
Clinical Symptoms				
S1	Over sedation\hypotension\falls^9^	6	2	33.3
S2	Rash^9,24^	285	35	12.3
Intervention Measures				
T1	Abrupt medication stop^6^	15	13	86.7
T2	Transfer to higher level of care^10,20^	15	4	26.7
T3	Rescue^20^	27	7	25.9
	Total	2291	304	13.3

^†^ADEs, Adverse drug events; it indicates the number of adverse drug events detected by triggers, and different triggers in a patient can indicate same adverse drug events.

^‡^PPV, Positive predictive value; it indicates the percentage of triggers that predict ADEs.

### ADE Characteristics

A total of 247 ADEs (Table 3) were identified in 221 patients (12.7%), 80.2% (198/247) occurred during hospital stays, and 19.8% (49/247) pre-existed as the reasons for the hospital admission: 204 (82.6%) were determined to be the National Coordinating Council for Medication Error Reporting and Prevention (NCC MERP) harm category E, 29 (11.7%) were category F, and 14 (5.7%) were category H.

**Table 3:** 247 cases of ADEs Classes.

Organ /System	ADE	No.	Total No. (%)
Gastrointestinal disorders	Diarrhea	34	76 (30.8%)
	Constipation	21	
	Vomiting	21	
Central and peripheral nervous system disorders	Convulsions	4	52 (21.1%)
	Convulsions grandmal	1	
	Over sedation\Hypotension	47	
Skin and appendages disorders	Rash	35	35 (14.2%)
Resistance mechanism disorders	Candidiasis	25	27 (10.9%)
	Infection fungal	2	
Metabolism and nutrition disorders	Hyperkalemia	1	18 (7.3%)
	Hypokalemia	6	
	Hypoglycaemia	5	
	Hyperglycemia	5	
	Hyponatremia	1	
Liver and biliary system disorders	Hepatotoxicity/Increased transaminases	18	18 (7.3%)
Platelet, bleeding and clotting disorders	Coagulopathy	4	4 (1.6%)
Respiratory system disorders	Respiratory depression	2	4 (1.6%)
	Bronchospasm	1	
	Dyspnoea	1	
White cell disorders	Leukopenia	3	3 (1.2%)
Musculoskeletal disorders	Dystonia	2	3 (1.2%)
	Arthritis	1	
Body as a whole-general disorders	Allergic reactions	1	2 (0.8%)
	Anaphylactoid reaction	1	
Urinary system disorders	Nephritis/Nephrosis	2	2 (0.8%)
Psychiatric disorders	Euphoria	1	1 (0.4%)
Heart rate and rhythm disorders	Tachycardia	1	1 (0.4%)
Other		1	1 (0.4%)
Total		247	247 (100)

The calculated rates of these 247 ADEs based on 1746 patient records were 13.7 (95% CI 11.2 to 16.1) ADEs per 100 patients, 17.4 (95% CI 14.1 to 20.7) per 1000 patient days, 1.2 (95% CI 1.0 to 1.4) per 1000 doses, and 9.9 (95% CI 8.3 to 11.5) per 1000 drugs.

### Risk factors associated with the occurrence of ADEs

Univariate analysis showed that the length of hospital stay, antibacterial use, numbers of drugs, doses and admissions were risk factors for the occurrence of ADEs (Table 1). Logistic regression results showed that only numbers of drugs, doses and admissions had a statistical significance (*p* < 0.05), while antibacterial use (*p* = 0.957) did not. Among them, the length of hospital stay was a protective factor (β = −0.101), for it did not in accord with the report in the literature^19^ (Table 4). This may not be independent of the various risk factors, but there is a certain linear correlation, namely multicollinearity.

**Table 4:** Logistic regression results of risk factors for the occurrence of ADE.

Variables	β	SE	Wald	Odds ratio	95% CI	P
Length of hospital stay	−0.101	0.026	15.218	0.904	0.859–0.951	0.000
Number of drugs	0.077	0.015	27.407	1.080	1.049–1.112	0.000
Number of doses	0.007	0.001	25.265	1.007	1.004–1.009	0.000
Number of admission	0.099	0.048	4.331	1.104	1.006–1.212	0.037
Antibacterial use	0.009	0.169	0.003	1.009	0.725–1.404	0.957
	−3.385	0.234	209.296	0.034		0.000

Multicollinearity diagnostic results showed that the largest condition index was 9.932, and the variance were 37%, 48%, and 68% for the length of hospital stay, number of drugs, and number of doses, respectively, suggesting collinearity (Table 5).

**Table 5:** Collinearity diagnostics results of the factors associated with the occurrence of ADEs.

Dimension	Eigenvalue	Condition Index	Variance Proportions					
			Constant	Length of hospital stay	Number of drugs	Number of doses	Number of admission	Antibacterial use
1	4.792	1.000	0.00	0.00	0.00	0.00	0.01	0.01
2	0.487	3.137	0.02	0.02	0.00	0.04	0.49	0.03
3	0.391	3.502	0.00	0.03	0.00	0.03	0.01	0.80
4	0.198	4.923	0.24	0.00	0.06	0.12	0.38	0.10
5	0.084	7.554	0.05	0.58	0.45	0.12	0.00	0.05
6	0.049	9.932	0.69	0.37	0.48	0.68	0.11	0.00

ADEs as a dependent variable, the number of drugs, doses, admissions and antibacterial use were screened into multivariate analysis. The significant factors associated with the occurrence of ADEs were the number of drugs, number of doses and number of admission (Table 6).

**Table 6:** Stepwise logistic regression results of the occurrence of ADEs.

Variables	Odds ratio	95% CI	P
Numbers of drugs	1.071	1.042–1.100	0.000
Number of doses	1.003	1.001–1.005	0.001
Number of admissions	1.115	1.016–1.224	0.022

## Discussion

We established 31 kinds of pediatric-focused triggers of ADEs. We increased the case of ‘A9 Nystatin and Sodium bicarbonate use’ and did not include the case of use for Vitamin K, which was different from the prior study. This may be relate to the fact that many people use Vitamin K for children post-operative supplement, ease bronchospasm and so on perhaps owing to cultural background. Thus, cases of use for Vitamin K were included, which led to many false positive results.

Our overall PPV of the trigger tool was 13.3%, within the range of other trigger tools in pediatric care from 3.7% to 38%^9–12,20,21^ that might reflect variations in practice. Among the 31 kinds of triggers, substantial differences in the PPV of individual triggers were found. Some triggers had shown a high PPV of >50%, such as ‘A2 Flumazenil use’,‘A9 Nystatin and Sodium bicarbonate use’, and ‘T1 Abrupt medication stop’; whereas four frequently identified triggers (≥300) had very low PPV, which might be due to the following reasons. First, in cases of anti-emetic use, false positives might have resulted from the common prophylactic use of anti-emetics in chemotherapy and post-operative care. Second, laxatives were often administered in patients with invasive procedures or surgical operations for preoperative preparation. Third, for the trigger ‘A1 Anti-allergic or Adrenaline use’ and ‘A7 Antidiarrheal or Antidiarrheal administration’, these drugs were commonly used for disease treatment as well as ADEs, leading to false positive results. Therefore, these triggers should be further specified for their use in treatment of disease or ADEs. In addition, some of the triggers were not identified in the review process such as blood drug concentrations, likely due to fewer pediatric patients on treatment or lack of monitoring.

The incidence of pediatric ADEs in this study was found to be similar to or lower than those found in other studies: Takata *et al*. reported 15.7 ADEs per 1000 patient-days^9^; Sakuma *et al*. reported 37.8 ADEs per 1000 patient-days^4^. These differences may reflect variations in local practices and study subjects. Among all ADEs identified in this study, there was no category G or I, and 94.3% were identified as temporary harm to the patient. This finding may be partially due to the short study duration and also explained by the fact that patients were excluded in the Hematology and Oncology, pediatric intensive care unit (PICU) or Neonatal Ward patients.

The risk factors for ADEs in pediatric inpatients included, but not limited to, the number of drugs, the number of doses, and the number of admissions in our study. Gender and age were not associated with the occurrence of ADEs in this study. This was consistent with previous findings^17,22^. A study showed that age is not an independent risk factor of ADEs, older children were likely to experience ADEs has been shown to be associated with they have more opportunities for the use of high-risk drugs^17^.

The increase in the number of drugs was most frequently found to be independent risk factors for ADEs. A systematic review of 26 studies with a total of 85212 patients confirmed that the number of drugs was an independent risk factor for ADEs^14^. This may be due to the additional risk of an ADEs when receiving several drugs, to drug-drug or drug-disease interactions, and to greater susceptibility of medication errors during hospital stay. Patients at an increase in the number of doses were more likely to experience ADEs, however additional studies are required to identify the risk factor for ADEs.

The number of admissions as a risk factor might due to the use of high-risk drugs. The children with more number of admissions in our study were mostly diagnosed with epilepsy, kidney disease, diabetes and other chronic diseases or recurrent infection, and for the treatment of specialist drug, such as anti-epileptics, systemic corticosteroids, immunosuppressive agents, analgesics, and antibacterial have been shown to be high risk for ADEs in hospitalized children by Rashed *et al*.^17^. Single factor analysis showed that the use of antibiotics was a risk factor for ADEs, while there was no significant difference between the stepwise logistic regression analysis, we could not rule out that it was not associated with the occurrence of ADEs.

The length of hospital stay as a risk factor for ADEs is controversial. Some studies have been considered that the length of hospital stay was a risk factor for ADEs, while others considered ADEs could be a cause of longer length of stay^17,22^. Length of hospital stay was significantly associated with the occurrence of ADEs, but the causal relationship need more research to confirm. Patients who stayed longer might have a need for more drugs, so they might have more opportunities to experience ADEs, while ADEs might result in a longer hospital stay. The number of drugs was an independent risk factor, and there was a linear relationship with the number of doses and the length of hospital stay, which may partly explain the relationship between hospital stay and ADEs. In line with Asia *et al*., we considered the length of hospital stay as a consequence of having an ADE but not as a risk factor for ADEs^17^.

This study showed that the IHI GTT was a useful method for the detection of ADEs in a Children’s hospital in China. However, there were some limitations. First, the 31 kinds of pediatric-focused triggers of ADEs could be improved to be more specific. Second, the triggers should be further tested in more pediatric including those in the Hematology and Oncology, PICU and Neonatal Units.

## Conclusion

To our knowledge, this was the first study to investigate ADEs of pediatric inpatients using GTT in China. More than one fifth of the pediatric inpatients experienced at least one ADEs, and most of the experiences caused temporary harm. The most significant factors of ADEs included the number of drugs, the number of doses and the number of admissions. In addition, triggers that had high PPV could be incorporated into routine screen systems to improve inpatient safety in the future.

## Methods

### Study design, setting and sample

This study employed retrospective medical record reviews. We conducted this study in the CHCMU, a large tertiary teaching children’s hospital in China. The hospital had 1400 beds. The number of inpatients was 65 thousand in 2014 and that of outpatients and emergency patients was 2.18 million annually. The hospital has fully electronic medical record and bar code systems for administering medications.

The sample size was 600 cases per year. It was determined on the basis of the prior studies indicating that the rate of pediatric inpatients ADEs was about 15%. The annual number of inpatients set a precision of ±3% with a 0.05 probability of type 1 error, after it was considering not conformed to the standard records we appropriately expand.

### Study source

Medical record management system was used to extract medical record conformed to the following standard patients from January 1, 2013 to December 31, 2015. Eligible patients were more than 28 days old and less than 18 years old, with a length of more than one days hospital stay and were discharged or died between January 1, 2013 and December 31, 2015. Patients were excluded if they were without drug exposure or when they were in PICU, in neonatal ward, in Hematology and Oncology, in the day hospital or observation unit. Random equidistant sampling method was used for sampling. A sample of 50 patients was randomly selected from standard patients monthly, which began in January 2013, with a total of 1800 patients.

### Triggers

The trigger items of CHCMU were developed based on 51 kinds of triggers recommended in ‘IHI GTT for Measuring Adverse Events’ and previous trigger tool studies by using triggers. Through the preliminary experiment and consultation, the experts determined the trigger list.

### Records Review

We employed a three-stage review process for medical records. In the first stage, one pharmacist reviewed each medical record for the presence of any of the triggers with a limit of no more than 20 min per chart. The medical records were reviewed in the following order: diagnoses and treatment procedures, discharge summaries, medication charts, laboratory results, operation notes, nurse notes, physician notes and admission note. The basic information of patients was recorded with the identified trigger, which led to a further review to determine the occurrence of ADEs.

In the second stage, two pediatricians reviewed all the medical records with identified triggers from the first stage to determine the presence of ADEs, its category and severity. If there was a disagreement, the final decision was made on the basis of a consensus at the research team meetings.

In the third stage, the pharmacist entered the data of the medical record review. All reviewers were required to read the white paper of the ‘IHI GTT for Measuring Adverse Events’ and trained in records review methods before the review.

We focused on ADEs that cause actual patient harm, but not medical errors that has potentials for patient harm. Harm was defined as an unintended physical injury resulting from or contributing to medical care that requires additional monitoring, treatment or hospitalization, or that results in death. Once harm was identified, its severity was evaluated by using the classification from the NCC MERP Index for Categorizing Errors^6^. It included temporary harm to the patient and required intervention (category E), temporary harm to the patient and required initial or prolonged hospitalization (category F), permanent patient harm (category G), required intervention to sustain life (category H), and the patient’s death (category I).

### Statistical Analysis

Data were analyzed by using Microsoft Excel 2011 and SPSS 23.0 software. We calculated ADEs per 100 patients admissions, ADEs per 1000 patient days, ADEs per 1000 doses and ADEs per 1000 drugs. Comparisons between groups were made by using the χ^2^ test for categorical variables and *t* test for continuous variables. *P* Values lower than 0.05 were considered significant. Stepwise logistic regression was used to investigate risk factors associated with ADEs. Multiple collinearity diagnosis was performed by variance decomposition proportion.

### Ethics Statement

This study was exempt from ethical review by the Institutional Review of the Children’s Hospital of Chongqing Medical University. Its compliance with the Ministry of Health’s 2007 Chinese Regulation on Ethical Review of Biomedical Research Involving Human Subjects. The methods were carried out in accordance with the relevant guidelines and regulations.
